# Circadian genomics of the chick pineal gland *in vitro*

**DOI:** 10.1186/1471-2164-9-206

**Published:** 2008-05-03

**Authors:** Stephen P Karaganis, Vinod Kumar, Phillip D Beremand, Michael J Bailey, Terry L Thomas, Vincent M Cassone

**Affiliations:** 1 Department of Biology, Texas A&M University, College Station, TX 77843-3258, USA; 2 Department of Zoology, University of Lucknow, Lucknow 226007, India; 3 Department of Poultry Science, Texas A&M University, College Station, TX 77843-3258, USA

## Abstract

**Background:**

Chick pinealocytes exhibit all the characteristics of a complete circadian system, comprising photoreceptive inputs, molecular clockworks and an easily measured rhythmic output, melatonin biosynthesis. These properties make the *in vitro *pineal a particularly useful model for exploring circadian control of gene transcription in a pacemaker tissue, as well as regulation of the transcriptome by primary inputs to the clock (both photic and noradrenergic).

**Results:**

We used microarray analysis to investigate the expression of approximately 8000 genes within cultured pinealocytes subjected to both LD and DD. We report that a reduced subset of genes was rhythmically expressed *in vitro *compared to those previously published *in vivo*, and that gene expression rhythms were lower in amplitude, although the functional distribution of the rhythmic transcriptome was largely similar. We also investigated the effects of 6-hour pulses of light or of norepinephrine on gene expression in free-running cultures during both subjective day and night. As expected, both light and norepinephrine inhibited melatonin production; however, the two treatments differentially enhanced or suppressed specific sets of genes in a fashion that was dependent upon time of day.

**Conclusion:**

Our combined approach of utilizing a temporal, photic and pharmacological microarray experiment allowed us to identify novel genes linking clock input to clock function within the pineal. We identified approximately 30 rhythmic, light-responsive, NE-insensitive genes with no previously known clock function, which may play a role in circadian regulation of the pineal. These are candidates for future functional genomics experiments to elucidate their potential role in circadian physiology. Further, we hypothesize that the pineal circadian transcriptome is reduced but functionally conserved *in vitro*, and supports an endogenous role for the pineal in regulating local rhythms in metabolism, immune function, and other conserved pathways.

## Background

The avian pineal gland serves as part of a multi-oscillatory circadian system [1] and influences other oscillators and downstream processes at least in part via its circadian secretion of melatonin [1-5]. At the cellular level, the avian pineal gland contains all the components needed for a functional circadian system as it possesses photoreceptors enabling direct entrainment to light [6-8], it contains a circadian oscillator [9,10], and it produces a measurable molecular output in the form of rhythmic melatonin biosynthesis and secretion [10,11]. These processes are properties of pinealocytes themselves since they continue *in vitro *as well as *in vivo *[8-10].

The pineal glands of several species of birds rhythmically synthesize melatonin over multiple circadian cycles in both organ culture and dispersed cell cultures, under constant darkness or dim red light [9-13]. The biosynthetic pathway for melatonin synthesis has been well characterized, involving four enzymatically catalyzed reactions to produce melatonin from the amino acid tryptophan [14]. The mRNAs for *TrH*, *AANAT *and *HIOMT *are rhythmically expressed in the chick pineal gland *in vivo *and *in vitro*, and rhythmic post-transcriptional regulation of these enzymes has been demonstrated [15-19], suggesting that the circadian clockworks within pinealocytes regulates this process at multiple levels of cellular organization.

The mechanism linking the core circadian oscillator(s) in pinealocytes with the melatonin biosynthetic machinery, on the other hand, is not completely understood. As stated above, pinealocytes respond directly to light *in vitro*, and there are at least three separable pathways by which light affects melatonin levels: 1) acute suppression of melatonin synthesis, 2) decrease in rhythm damping and 3) phase shifting of the circadian pacemaker underlying melatonin rhythms [12]. The acute effects of light are mediated, at least in part, by a reduction in cAMP levels, which leads to a decrease in AANAT protein levels as well as a modest decrease in *AANAT *transcription [2,3,12,17,20,21].

In the chick, norepinephrine (NE) also effects an acute inhibition of melatonin biosynthesis through a reduction in intracellular cAMP levels [6,22]. Similarly, daily light and/or NE administration decreases damping of the rhythm of melatonin release via a cAMP-dependent pathway [23,24]. Thus, it appears that light and NE influence pineal output in multiple ways through a common signal transduction pathway. NE does not, however, exert any phase-shifting effects on melatonin biosynthesis rhythms, and therefore sympathetic input, unlike light, does not serve as a Zeitgeber for the chicken pineal clock [2]. The cellular pathway(s) underlying phase shifting of the pineal oscillator does not involve cAMP signal transduction [2,3,20], and remains unresolved at this time.

The molecular basis of the circadian clock mechanism itself is poorly understood in birds, although avian orthologs of most canonical clock genes (i.e., genes thought to comprise the molecular oscillator in mammalian clocks) have been isolated, cloned and characterized [25,26]. However, the dynamic interactions of these genes and their products have not been systematically studied in as much detail as it has been in mammals. In mammals, the clock mechanism is thought to consist of interlocking feedback loops of "positive" and "negative" clock gene elements, which are regulated at the transcriptional and translational levels, as has been elegantly demonstrated in *Drosophila *and other model systems [27-30]. It is unknown which components of this system are functionally conserved in avian species.

Previously, our laboratory has utilized high-density cDNA microarray technology to obtain a transcriptional circadian profile of approximately 8,000 pineal-specific chick cDNAs expressed *in vivo *within the pineal gland and retina [25,26]. This research has revealed a complex circadian orchestration of a diverse array of pineal transcripts, including "clock gene" orthologs, photo-transduction components, immune function genes, and protein processing and trafficking components. Here, we apply our genomic approach to the study of the chick pineal gland *in vitro*, and utilize a unique screen to identify novel genes involved in clock function. Based upon our previously published data [25,26] and published reports [2-12], we hypothesize that central clock mechanisms in the chick pineal gland are likely identical or, at least, very similar in the retina and must be retained *in vitro*. Further, light should affect expression of these genes, while norepinephrine should only affect output. Therefore, we utilized the pineal-specific microarray developed and used in previous studies from our laboratory [25,26] to identify genes that met the following criteria in cultured pinealocytes: 1) exhibit a rhythmic mRNA expression pattern that persists in constant darkness; 2) are light responsive; and 3) are insensitive to NE administration. These should represent a subset of genes identified in both pineal gland and retina *in vivo *[25,26].

## Results

### Pineal melatonin rhythms

**Figure 1:**
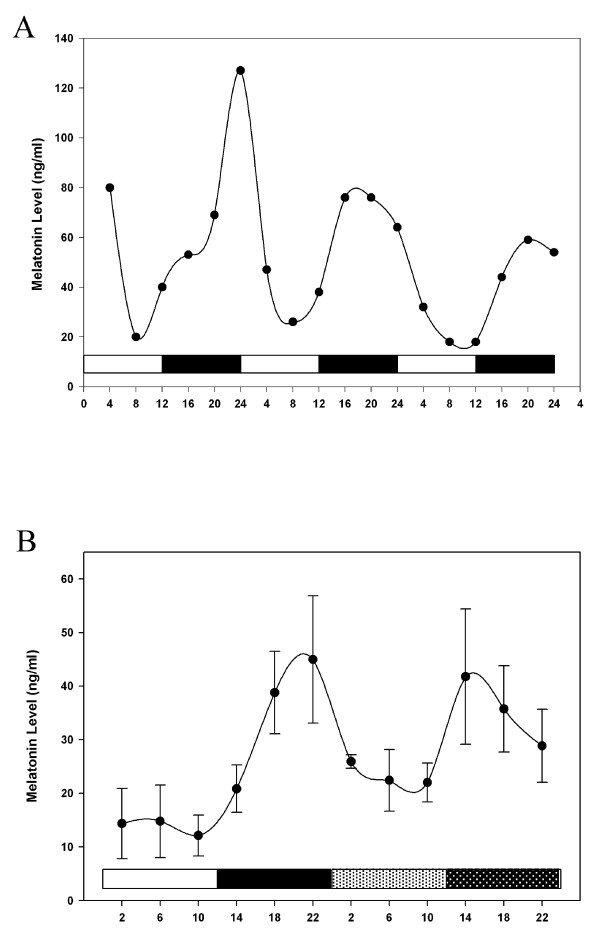
**Pineal melatonin rhythms**. **A**. Levels of melatonin secreted by chick pinealocytes were measured for 3 days in culture under an LD cycle. White bars indicate the time when lights were on, and black bars indicate the time when lights were off. **B**. Melatonin levels were measured from cultured pinealocytes maintained one day of LD followed by one day of DD. Light hatched bars indicate subjective day, while dark hatched bars indicate subjective night.

We measured melatonin secretion by the pineal cultures to monitor physiological output in parallel with the gene expression analysis. Initial pilot studies performed demonstrate the *in vitro *pineal cultures are capable of entrainment to a LD 12:12 cycle. As expected, cultured pinealocytes exhibited rhythmic melatonin production for at least three days in a LD 12:12 cycle (Fig. 1A), with a phase consistent with previous reports. The melatonin rhythm of pinealocytes used in the array analysis persisted in constant darkness with reduced amplitude (Fig. 1B).

### Rhythmic transcriptome

In order to select statistically significant rhythmic genes while excluding erratically expressed genes, we used two different statistical filters to screen for rhythmicity (as described in the methods), and present the data here as discrete data sets (Additional files 1, 2). All sequences, BLAST results, and alignments are listed by reference number and are accessible through the Texas A&M Biology Department's Laboratory for Functional Genomics chicken pineal database [31].

Using the t-test comparison method, we found that 446 (5.5%) of the cDNAs represented on the array exhibit at least a 1.5-fold amplitude rhythm in LD (Additional file 1). Of these, 191 were unique, classified genes, 216 returned no BLAST hit, and the remainders were redundantly represented cDNAs. The genes showing the greatest redundancy in this data set were *HIOMT *(n = 10), *TrH *(n = 9), *transthyretin *(n = 8), *cystatin c *(n = 5), and *purpurin *(n = 4). The total number of transcripts showing 2-fold rhythmic expression was greatly reduced, representing only 76 genes, or 0.9% of all genes on the array. Of these, 18 were unique, classified genes, while 44 were unknown, with the remainder being redundancies. Not surprisingly, most of the redundant cDNAs were *HIOMT *(n = 7), *TrH *(n = 6), and *purpurin *(n = 4).

Applying the same statistical method to the DD data set, we found that 337 cDNAs (4.2%) exhibit at least a 1.5-fold-amplitude rhythm in DD (Additional file 1). 150 of these were unique, classified genes, 164 were unknown, and the remainders were redundant transcripts. The reduced number of redundant, rhythmic genes in the DD data set likely indicates that some cDNAs, although rhythmic, did not meet our 1.5-fold change criterion. This is supported by the fact that overall transcriptional rhythmicity, and to a lesser extent, melatonin production, was reduced in DD. In fact, only 33 total cDNA's showed at least a 2-fold change in expression in DD, of which 14 were unique, classified genes, and 15 were unknown.

Using ANOVA as a statistical filter, the total number of rhythmically expressed transcripts with a 1.5-fold or greater amplitude in LD was reduced to 187 (2.3%), representing 71 unique, classified genes and 91 unidentified transcripts (Additional file 2). The most commonly repeated cDNAs were again *HIOMT *(n = 9), *TrH *(n = 8), *transthyretin *(n = 5), and *purpurin *(n = 4) with *cystatin c *only being represented twice. While the t-test method was more inclusive overall, 11 out of the 71 classified genes which passed the ANOVA filter alone did not pass the t-test filter. Screening for 2-fold rhythmic expression using the ANOVA statistical filter reduced the list to 76 total genes (0.9%), including 26 unique, classified genes and 39 unknown transcripts. The disparity in the number of genes showing 2-fold or greater rhythmicity in LD as reported using the two different statistical filters was quite low. This was not unexpected, given that genes cycling with higher amplitude are more likely to show statistical significance using either method. However, 10 of the combined 44 classified genes within the two gene lists were mutually exclusive.

**Figure 2:**
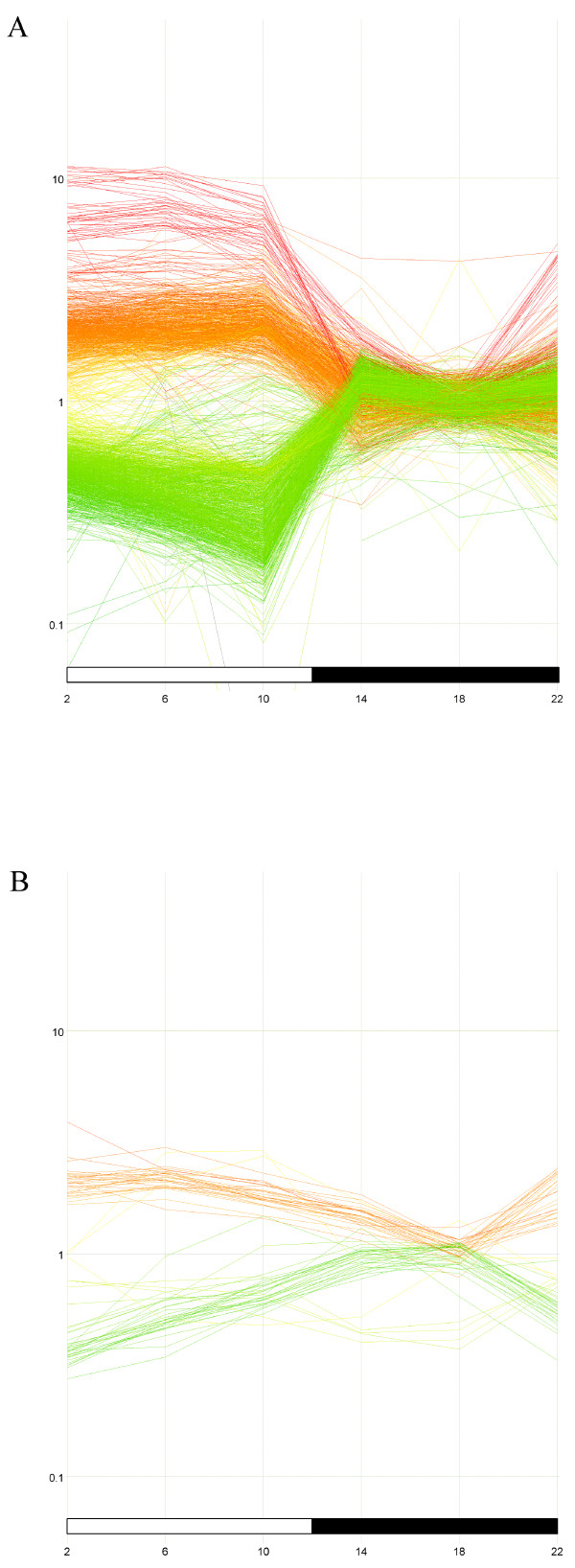
**Rhythmic transcripts *in vivo *and *in vitro***. Gene expression profiles are shown for transcripts which cycle with a 2-fold rhythm in pineal in vivo (A) or in cell culture (B) under LD conditions. Statistical filtering of each data set is based on ANOVA as well as t-tests for the cell culture experiment.

Using the ANOVA-based statistical analysis for the DD data set, we found that a total of 108 (1.3%) transcripts, including 47 unique, classified genes and 54 unidentified transcripts exhibited a 1.5-fold amplitude rhythm (Additional file 2). 17 out of 47 of the classified genes did not pass the t-test filter. Only 22 total transcripts passed our ANOVA-based screen at the 2-fold level, with 11 unique, classified genes and 10 unknown transcripts. However, 15 out of the combined 25 classified genes within these two gene lists were mutually exclusive. Overall, our pineal cultures showed a large reduction in the number and amplitude of rhythmic transcripts compared to what has been observed *in vivo *(Fig. 2). In spite of this result, the amplitude of the melatonin secretion rhythm was robust and comparable to that observed in serum of chicken *in vivo *[32].

### Rhythmic functional gene groups

**Figure 3:**
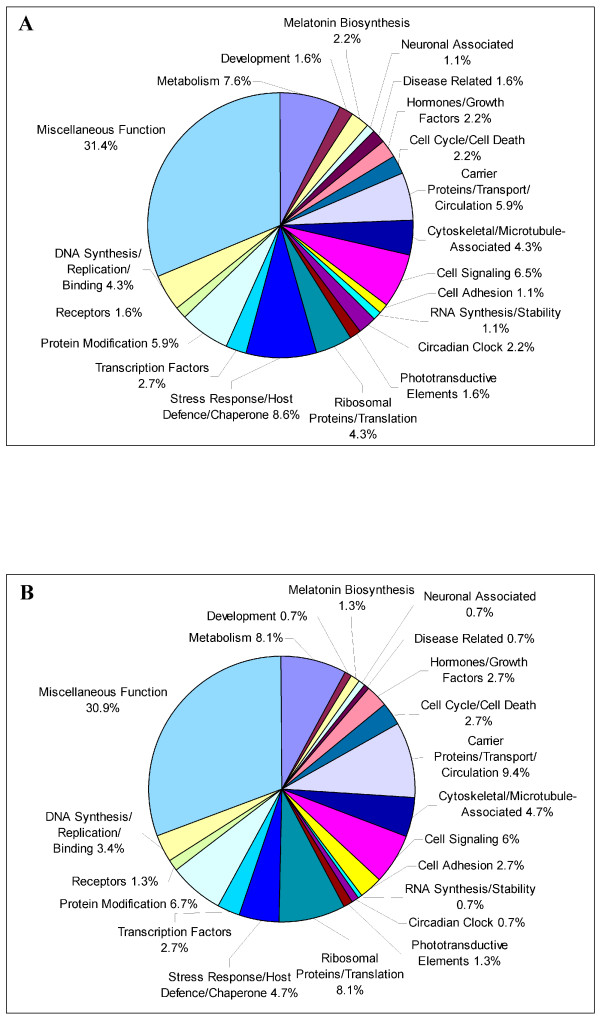
**Rhythmic gene functions**. Rhythmically transcribed genes were clustered according to proposed function, and the percentage of rhythmic genes representing each category is given under LD (**A**) or DD (**B**) conditions.

Genes that exhibited 1.5-fold rhythmic expression in LD or DD were classified into one of twenty-one different functional categories using the same schema published previously in our laboratory [25,26]. This type of analysis permits a comparison of pineal transcriptome regulation *in vivo *and *in vitro*. We performed this analysis on the data set from the t-test based analysis, reasoning that the larger data set would minimize the possibility of sampling error. In both LD and DD, the functional groups exhibiting the largest degree of circadian regulation were those associated with protein modification, intermediary metabolism, stress-response/immune function, cellular signaling, transport, and ribosomal proteins/translation (Fig. 3; Additional file 3).

### Array validation of selected genes

**Figure 4:**
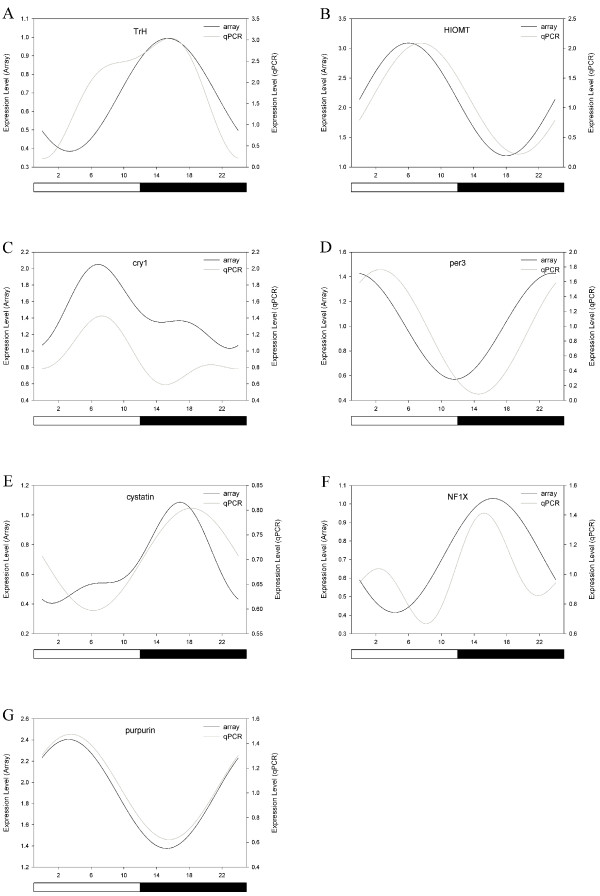
**Microarray validation**. qPCR was used to validate rhythmic expression of the following genes under LD conditions: TrH, HIOMT, cry1, per3, cystatin c, NF1X, and purpurin (A-G, respectively). Cosinor functions fitted to data from microarray analysis using GeneSpring output (black lines) and from qPCR analysis (grey lines) are plotted. Cosinor analysis and ANOVA were performed on each data set. White bars indicate lights on, and black bars indicate lights off. Light hatched bars indicate subjective day, while dark hatched bars indicate subjective night.

To validate the experimental data, the mRNA expression levels of four well characterized genes in the chick pineal gland were analyzed using qPCR techniques. Two genes from the melatonin biosynthesis pathway (*TrH *and *HIOMT*) and two clock genes (*cry1 *and *per3*) were chosen for validation under LD conditions. Corroborating the microarray data, melatonin biosynthesis genes exhibited high amplitude circadian rhythms when measured using qPCR. *TrH *expression was rhythmic (array p_cosinor _< .001; array p_ANOVA _< .001; qPCR p_cosinor _< .001; qPCR p_ANOVA _< .001), with > 2-fold higher mRNA levels at night (Fig. 4A). As expected [16], *HIOMT *expression was approximately antiphase to the *TrH *rhythm, peaking at midday, with a large (~3-fold) amplitude rhythm in LD (array p_cosinor _< .001; array p_ANOVA _< .001; qPCR p_cosinor _< .001; qPCR p_ANOVA _< .001) (Fig. 4B).

The amplitude of clock gene rhythms was reduced compared to those of the melatonin biosynthesis genes. *Cry1 *expression was rhythmic (array p_cosinor _< .001; array p_ANOVA _< .001; qPCR p_cosinor _= .001; qPCR p_ANOVA _= .003) with peak expression occurring at ~ZT6 (Fig. 4C). P*e*r3 mRNA expression was rhythmic in LD (array p_cosinor _< .001; array p_ANOVA _< .001; qPCR p_cosinor _< .001; qPCR p_ANOVA _< .001), with peak expression occurring between ZT22-2 (Fig. 4D). The phases of the rhythms of both clock genes, as well as the melatonin biosynthesis genes, were similar when measured using either qPCR or microarray hybridization techniques.

Additionally, we validated the expression of three genes of interest (*cystatin c*, *NF1X*, and *purpurin*) which were identified in our screen (see below). While temporal expression patterns of the genes have not been previously characterized in chick pineal, our microarray analysis reveals they exhibit circadian rhythms *in vitro. Cystatin c *exhibited higher expression at night, with a peak occurring around ZT18 as measured using either method (Fig. 4E), although qPCR did not show a significant change in expression using ANOVA (array p_cosinor _< .001; array p_ANOVA _< .001; qPCR p_cosinor _= .043; qPCR p_ANOVA _= .201). Microarray analysis and qPCR revealed a peak in *NF1X *expression between ZT14-ZT18 (Fig. 4F), although this rhythm was not significant as measured by qPCR, likely due to the detection of a secondary peak at ~ZT2 (array p_cosinor _< .001; array p_ANOVA _< .001; qPCR p_cosinor _= .334; qPCR p_ANOVA _= .176). *Purpurin *expression was highly rhythmic, with identical phases measured using either method (Fig. 4G; array p_cosinor _< .001; array p_ANOVA _< .001; qPCR p_cosinor _< .001; qPCR p_ANOVA _< .001).

### Regulation by light and norepinephrine

**Figure 5:**
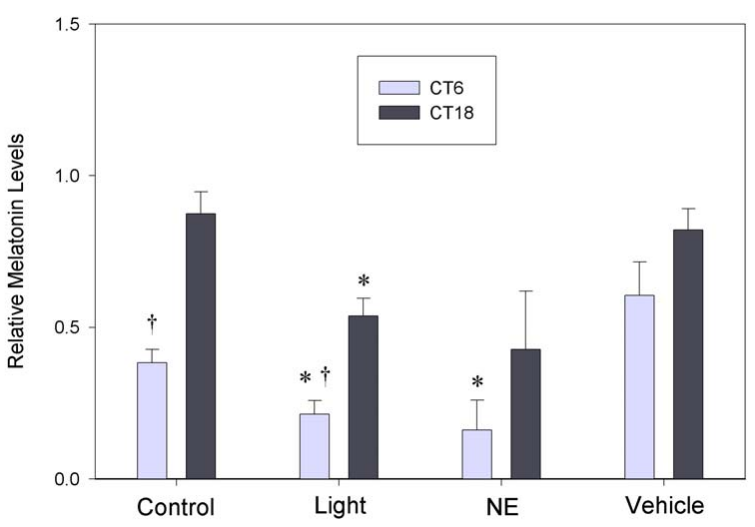
**Inhibition of melatonin production by light and norepinephrine**. Melatonin levels released into media were measured during mid-subjective day and mid-subjective night for cultures that had received a 6-hr light pulse, those that had received a 6-hr dose of NE (3 × 10^-8 ^M), and for control cultures which had received no light or a vehicle solution. Significant difference (p < .05) between experimental treatments and controls for each timepoint is indicated by *. Significant difference (p < .05) between CT6 and CT18 timpoints within each treatment group is indicated by †.

As expected, 6-hour exposure to a light pulse (38 μW/cm^2^) inhibited melatonin release from the cultured pinealocytes at both subjective midday and midnight (Fig. 5). Norepinephrine administration (3 × 10^-8 ^M) significantly decreased melatonin release during the subjective day but not during the subjective night (Fig. 5). A total of 142 (~1.8%) cDNAs were shown to be regulated at least 1.5-fold by light. 50 of these were unique, classified genes, 71 were unknown, and the remainders were redundant cDNAs (Additional file 4). The most abundant light regulated genes were *HIOMT *(n = 10), *TrH *(n = 8), *cystatin *(n = 3), and *purpurin *(n = 4). The only clock gene that was shown to be light regulated was *cry1*, which was up-regulated by light (at both CT6 and CT18), consistent with previously published data [33]. Only a small number of transcripts (n = 24) were regulated 2-fold, although these include all the above except purpurin (Additional file 4).

The phototransductive/photoregulatory elements shown to be affected by light were *purpurin *(p*urp*) and *early-undifferentiated retina and lens gene *(e*url*). Other phototransductive/photoregulatory genes represented on our array were rhythmic, but not acutely light-regulated, including *retinal fascin*, *interstitial retinol-binding protein 3 *(i*rbp*), and *transducin γ-subunit*. All of these but the last are rhythmic *in vivo *as well [25].

A light pulse applied to pinealocyte cultures during CT0–CT6 affected the expression of a larger number of transcripts than when applied during CT12–CT18, including both induction (CT6, n = 54; CT18, n = 32) and suppression (CT6, n = 50; CT18, n = 30) of specific genes (Additional file 4). The total number of genes influenced by light exposure, however, was similar within a given treatment. Norepinephrine administration had little overall effect on gene expression – only 19 cDNAs showed 1.5-fold regulation by NE (Additional file 5).

### Comparative analysis and candidate genes

As part of our screen to identify candidate genes that may play a role in pinealocyte clock function, we compiled non-overlapping unigene lists which fit into combinations of one or more of the following categories, based on t-test analyses: 1) rhythmic genes with 1.5-fold amplitude expression in LD; 2) rhythmic genes with 1.5-fold amplitude expression in DD; 3) genes regulated 1.5-fold by light; and 4) genes regulated 1.5-fold by norepinephrine (Additional file 6). A summary of the number of genes in each list, ranked in order of decreasing numbers, is displayed in Table 1.

**Table 1:** Comparative gene list

**Gene List**	**# non-redundant genes**
LD only	234
DD only	172
LD, DD	102
Light only	44
LD, Light	34
LD, DD, Light	27
NE only	14
DD, Light	8
LD, NE	3
DD, NE	1
LD, DD, Light, NE	1
LD, DD, NE	0
LD, Light, NE	0
DD, Light, NE	0
Light, NE	0

Clustered, non-overlapping unigene lists, ranked in order of decreasing gene number. Genes are clustered as follows: LD: rhythmic genes with at least 1.5-fold amplitude mRNA expression in LD; DD: rhythmic genes with at least 1.5-fold amplitude mRNA expression in DD; Light: gene mRNA regulated at least 1.5-fold by light; NE: gene mRNA regulated at least 1.5-fold by norepinephrine.

**Figure 6:**
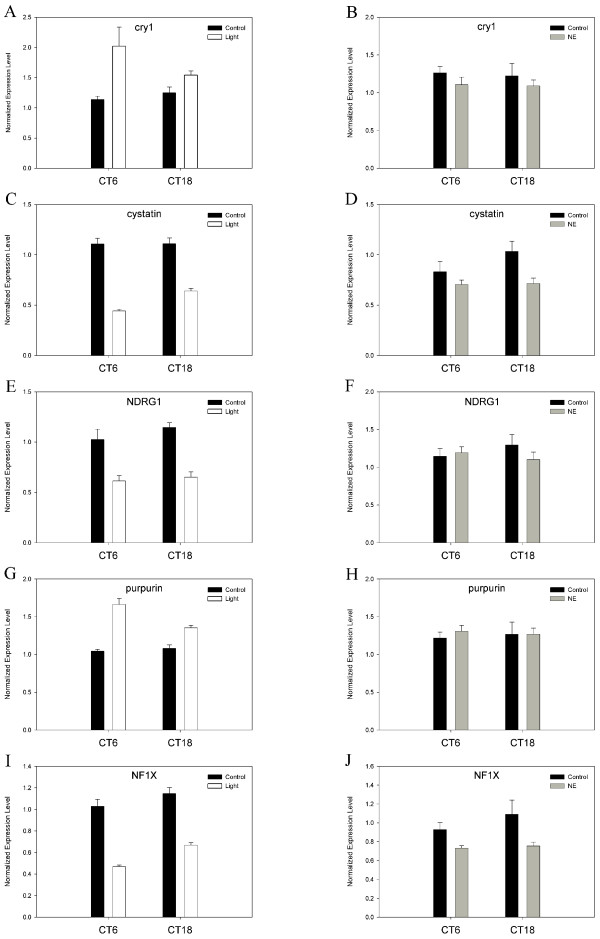
**Light regulated, NE-insensitive gene transcripts**. Expression data from selected genes that passed the criteria outlined in our screen are plotted here as histograms showing mRNA levels measured after receiving a 6-hour pulse of light (left panel) or 6-hour course of NE supplemented medium (right panel) relative to controls. Histogram plots are based on normalized array data from GeneSpring (Silicon Genetics).

**Figure 7:**
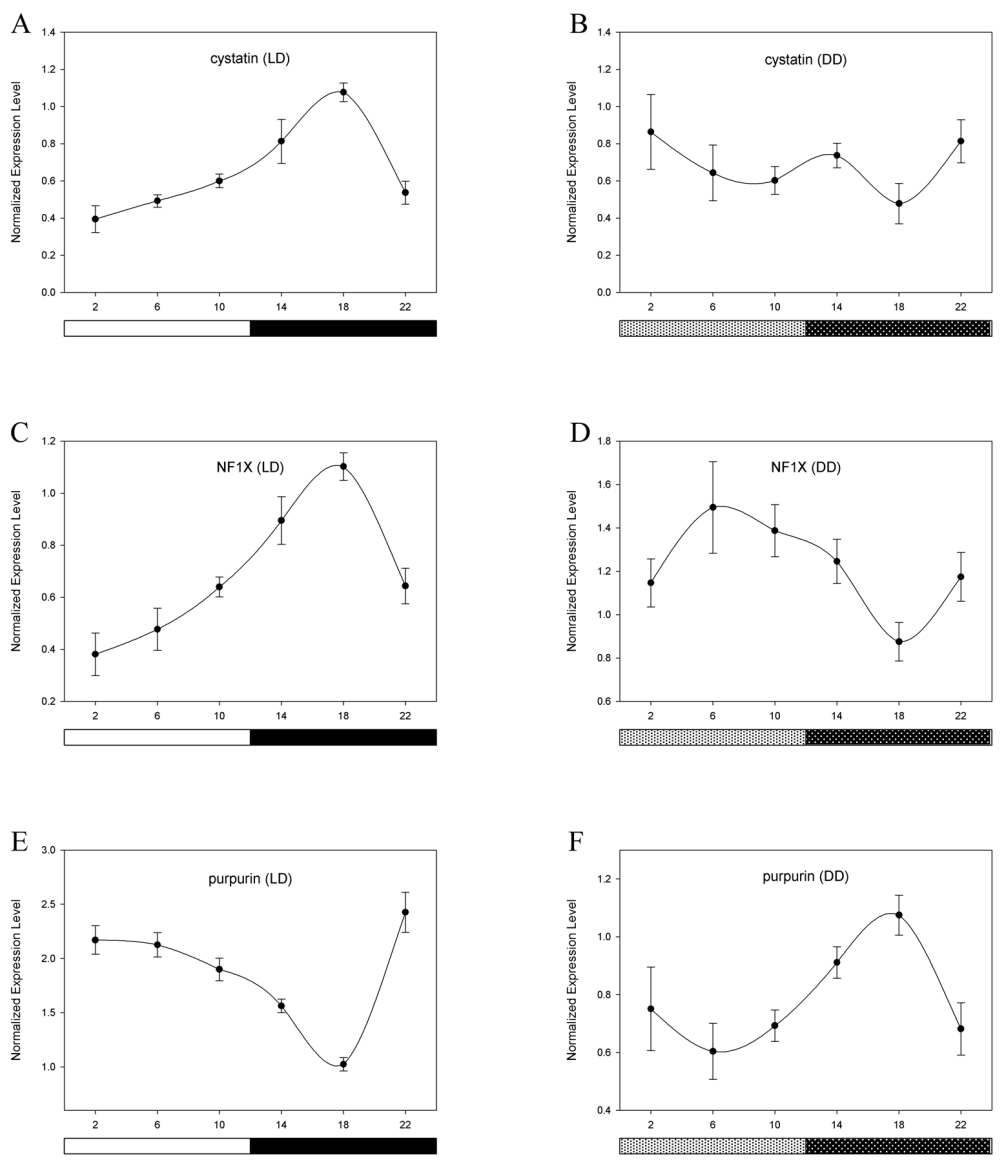
**Phase inversions of candidate gene mRNA rhythms in DD**. Circadian expression patterns of genes that exhibited phase inversions in expression rhythms when switched from LD to DD are shown here (LD, left panel; DD, right panel).

A nearly equal number of genes that were rhythmic in LD and affected by light were also rhythmic in DD. We consider those genes which met these criteria and were also unaffected by norepinephrine to be candidate "clock-related" genes requiring further analysis (Table 2; Fig. 6). Although *cry1 *did not continue to exhibit a significant rhythm in DD under our array analysis, we include it here because qPCR verifies that cry1 is in fact rhythmic under DD conditions (data not shown), and *cry1 *expression is potently induced by light (at CT6) but unaffected by NE at either timepoint (Fig. 6A–B). The mRNA rhythms of these genes under LD conditions correlate well with their regulation by light in all cases, as demonstrated for selected genes (Figs. 6, 7). However, some of these genes underwent a complete phase inversion in DD (Fig. 7).

**Table 2:** Candidate gene list

Cry1*‡
Cystatin c**‡
HIOMT**‡
N-myc downstream regulated 1*
Nuclear factor 1 × protein**‡
Hypothetical protein DaciDRAFT_2108 [Delftia acidovorans SPH-1]*‡
Purpurin **‡
PREDICTED: hypothetical protein [Gallus gallus] cp5764**‡
Proline-rich protein 15, isoform CRA_a [Rattus norvegicus]*
Unnamed protein product [Tetraodon nigroviridis]**‡
18 unidentified sequences

List of annotated genes that meet the following criteria: 1) they exhibit a rhythmic expression pattern that persists in constant darkness; 2) they are light regulated; and 3) they are insensitive to NE administration.

* Gene also passed ANOVA in one data set, either LD or DD

** Gene also passed ANOVA in both LD and DD data sets

‡ Gene is rhythmic *in vivo *in both LD and DD at the 2-fold level

## Discussion

Despite maintaining a robust rhythm of melatonin release comparable with previously reported rhythms *in vitro *[9,10] cultured pinealocytes exhibited lower amplitude mRNA rhythms within a diminished population of cycling transcripts as compared to what was reported *in vivo *[25]. Based on the two methods of analysis used in this study, our estimates of the number of genes expressing a 1.5-fold or greater rhythm within the pineal *in vitro *vary from ~2–6% of the genome in LD, and ~1–4% in DD, as represented in our array. Less than 1% of all pineal genes represented in our study express a 2-fold or greater amplitude rhythm in LD or DD using either method. While it is impossible to report the number of rhythmically expressed genes with absolute precision, it is likely that the actual proportion of rhythmic genes falls between our two estimates.

Nevertheless, it is clear that the chick pineal undergoes a large reduction in both the number of rhythmically transcribed genes and in the amplitudes of their rhythms *in vitro *as compared to *in vivo*, where a 2-fold or higher amplitude rhythm was observed for ~22% of the total number of transcripts in LD and ~8.5% of the total number of transcripts in DD [25]. This reduction in both the number of rhythmic transcripts and the amplitudes of the remaining rhythmic transcripts is not likely due to the failure of the microarray analysis, since exactly the same microarrays and microarray analyses were employed here as in two previously published *in vivo *analyses [26,27]. Further, it cannot be due to a reduction in the health of the pinealocytes, since a robust rhythm of melatonin release is retained in culture and persists in DD (Figure 1). This rhythm amplitude is comparable to plasma melatonin rhythms measured *in vivo *[32,34], and to similar *in vitro *pineal preparations [2,3,12]. This observation suggests that melatonin synthesis may be one of a small number of outputs from the circadian clock that continues to cycle at high amplitude in the absence of endogenous physiological feedback.

It is not surprising, then, that the largest and most consistent number of high-amplitude rhythmic transcripts were *HIOMT *and *TrH*, two genes involved in the melatonin biosynthesis pathway that are regulated by the circadian clock. Our array analysis did not show *AANAT *mRNA to be rhythmic in constant conditions as it is in other dispersed pinealocyte cultures [35], although this may be due to our placement of cells under constant darkness, as opposed to constant dim red light, as has been done in other studies utilizing the same culture system [35]. *In vivo*, the amplitude of *AANAT *is greatly reduced under DD conditions as well [25]. This may suggest that *AANAT*, despite being the rate-limiting enzyme in this pathway, may damp more readily in the absence of physiological stimuli such as norepinephrine, or that it is regulated primarily through post-transcriptional mechanisms. However, *HIOMT *and *TrH*, along with *cystatin*, *transthyretin*, and *purpurin*, had the most abundant number of rhythmic transcripts, consistent with observations *in vivo *[25].

The circadian phases of melatonin biosynthesis gene mRNA's are consistent with previous reports of mRNA regulation of these genes in the chick [25,26]. Orthologs of the clock genes *cry1 *and *per3 *also exhibited mRNA rhythms consistent with the literature and with their putative role as negative elements. It is worth mentioning that, although the canonical negative element clock genes were rhythmic, they oscillated with low amplitudes compared to many other genes represented on the array, especially genes involved in melatonin biosynthesis. Therefore, if the "clock genes" are driving all cellular mRNA rhythms, significant amplification steps must occur to produce the more robustly rhythmic outputs. Of course, we have not investigated rhythmicity at the protein level, and it is likely that post-transcriptional mechanisms play a significant role in the regulation of downstream processes by the clock.

Interestingly, the functional clustering of rhythmic genes in pineal culture is remarkably similar to what is observed in the pineal and retina *in vivo *[25,26], indicating that the reduction in the number of rhythmic genes in culture is global, rather than selective. The fact that pathways involved in immune-function are widely regulated by the pineal clock *in vitro *(see Fig. 3, Additional file 3) supports the notion that the pineal gland may play a more complex role in avian physiology than just the endocrine secretion of melatonin. While circadian control of these pathways may be specific to the pineal, it is also worth noting that genes involved in redox state/metabolism and protein processing appear to be highly regulated by the clock in other systems [30,36]. Thus, despite high specificity in circadian control at the level of the individual gene [30,36], many common functional outputs appear to be regulated by the clock across different species. Still, extra-pineal influences clearly play a significant role in driving many pineal rhythms, and therefore caution should be exercised when attributing mechanisms of these rhythms to endogenous pineal function.

Another intriguing observation is the large number of genes we found to be exclusively rhythmic in DD. Our broadest estimate indicates that as many as 73 (~50%) of the unique, classified genes found to be rhythmic in DD are not rhythmic in LD. Similar findings have been published from at least two other laboratories conducting array studies of *Drosophila *genomics [36-38]. One explanation for this phenomenon is that LD cycles could mask the rhythmicity of some light-regulated genes. However, we found very few genes that fit this profile, i.e., genes that were exclusively rhythmic in DD and were regulated by light (Table 1). Thus, another, unknown mechanism may exist where LD cycles indirectly inhibit rhythmic expression of some genes. Although these findings have been understated in the literature, we suggest that they are likely more than just an epiphenomenon, and may be an important, global aspect of the complex circadian orchestration of animal genomes.

The observation that light had a differential effect on mRNA levels at different times of day suggests that the pineal clock may modulate photo-responsiveness itself as a function of circadian time, such that light has a greater effect at a time when it is normally present as an exogenous stimulus. Although the amplitude of induction/reduction of mRNA by NE is small compared to a light stimulus, they have comparable effects on melatonin production. Thus, if NE has global effects on the chick pineal, it may exert its largest effects at the protein level, with comparatively small effects on gene expression, as is the case for NE's acute inhibition of melatonin biosynthesis [8].

Our comparative analysis revealed that many genes were rhythmic exclusively in LD or DD, or were rhythmic in both. As might be expected, there was significant overlap between genes that were rhythmic in LD only and those that were affected by a light pulse. The rhythmic expression of these genes is therefore probably light-driven, although some may have exhibited low amplitude rhythms in DD that were not detected on the array. Additionally, we found a significant number of genes that were regulated by light, but were not rhythmic in LD. Again, some of these genes may have expressed weak rhythms that went undetected. Another explanation is that light may be masking the endogenous rhythms of some of these genes in LD.

As noted in the results, some of these gene mRNA rhythms underwent a complete phase inversion in DD, suggesting that LD cycles may impose light-driven rhythms for some genes via acute inhibition/induction by light. Nevertheless, we cannot differentiate between acute and phase-shifting effects of light in this analysis, and therefore some of these genes may or may not fit the true criteria expected for clock-related genes. Also, because only one pass sequencing was conducted from the 5' ends of these genes, some of the "unknown" genes that did not return a significant BLAST hit might be identified with additional sequencing. Some of these may be redundant with other genes from our analysis. Several of these classified genes are rhythmic in chick retina as well [26], suggesting they may be a ubiquitous component of chick pacemaker tissues. Some of the most interesting classified candidates from our screen include: *NF1X*, a putative redox sensitive transcription factor; *cystatin c*, a cysteine protease inhibitor; *NDRG1*, a regulatory target of retinoic acid and proto-oncogenes; and purpurin, a lipocalin with putative immune or photoregulatory function. A brief discussion of these genes and their potential roles in pineal function is provided in Additional file 7.

## Conclusion

We reveal that pinealocytes, while maintaining robust circadian physiology, exhibit globally reduced transcriptional rhythms *in vitro*. This reduced subset is, however, reflective of the functional distribution of the larger rhythmic transcriptome *in vivo*. While chick clock gene orthologs continue to cycle in culture, they do so at low levels, suggesting that significant signal amplification and/or posttranscriptional regulation must occur if these genes are driving the larger amplitude rhythms seen in the physiological output of the cells, as well as the expression of other more highly rhythmic genes. Our experimental screen has provided a set of rhythmic genes that are sensitive to light, a potential phase-shift inducing stimulus, but not acute regulation by norepinephrine. This gene set supplies unique and intriguing candidates for deeper characterization of the circadian system, including knockdown and over-expression experiments that may lead to the identification of genes with novel circadian clock function in avian species.

## Methods

### Cell culture

All animals were treated in accordance with ILAR guidelines; these procedures have been approved by the Texas A&M University Laboratory Animal Care Committee (AUP no. 2001-163). One-day-old chicks were obtained from Hyline International (Bryan, TX), killed by decapitation, and their pineal glands were removed for cell culture following published protocols [12]. Briefly, excised glands were dispersed in trypsin, seeded into 12-well polystyrene tissue culture plates, and maintained in McCoy's 5A modified medium supplemented with 10% chicken serum, 10% fetal bovine serum, and 1% PSN antibiotic cocktail (Gibco/Invitrogen, Carlsbad, CA) in a humidified incubator at 37°C with 5% CO_2_. Cells were maintained on a 12-hour light: dark cycle (38 μW/cm^2 ^light intensity) for the duration of the culture, until sampling began. In order to maintain optimal growth rates and cell density, fetal bovine serum was left out of the culture medium on the second and third day. On the fourth day and thereafter, the cells were maintained in medium containing 10 mM KCl and no serum, as described previously [12].

### Experimental treatments and sampling

#### LD and DD experiment

On day 6 of the culture, cells were either kept in a 12 hour LD cycle or transferred to DD. Media was collected every four hours for a 24-hour period; sampling began 4 hours after lights on for cells in LD, or 4 hours after the beginning of the subjective light period for cells in DD. When sampling in the dark, infrared viewers were used. Media was pooled from all plates within each treatment, and stored at -20°C for melatonin RIA analysis. Cells from a single plate were harvested into Trizol reagent (Invitrogen) every four hours, beginning with ZT2 (in LD) or CT2 (in DD), in between time points during which media was being collected, i.e. at ZT/CT 2, 6, 10, 14, 18, 22. Trizol samples from each plate were immediately pooled, homogenized, and then frozen at -80°C for future RNA extraction. Four biological replicates were performed for each experimental timepoint.

#### Light pulse experiment

On day 6 of culture, cells were transferred to DD, and given a 6 hour light pulse (38 μW/cm^2^) from either CT12–18 or from CT0–6 the following day, a protocol known to be sufficient to elicit a phase shift in pinealocytes [2]. Control cultures were maintained in DD, and received no light pulse. At CT12 or CT0, cells were washed, the media was changed, and then collected at CT18 or CT6, respectively, for both control cultures and cultures that had received the light pulse. After media was collected, cells were harvested into Trizol, homogenized, and stored at -80°C. Four biological replicates were performed for each timepoint, for both light exposed and control treatments.

#### Norepinephrine experiment

The protocol used in this experiment was the same protocol used in the light pulse experiments, except experimental cultures received norepinephrine-supplemented media (3 × 10^-8 ^M) instead of a light pulse. Control cultures received media lacking norepinephrine. Four biological replicates were performed for each timepoint, for both treatments.

### Melatonin radioimmunoassay

Melatonin was measured using radioimmunoassay, which has been validated for chick plasma and cell culture medium [39]. Media samples were mixed with tricine buffered saline and incubated with 3H-radiolabeled melatonin (8,000–10,000 cpm per 100 μl) for 30 min. at room temperature. Samples were then incubated at 4°C overnight with sheep anti-melatonin antibody (Stockgrand Ltd., Surrey, UK) diluted to achieve on an optimal binding range of 20–25%. Bound melatonin was separated from free melatonin by addition of dextran-coated charcoal suspension and centrifugation at 4°C. Supernatant containing the bound antibody fraction was removed, placed into scintillant, and counted on a scintillation counter (Beckman Instruments Inc., Fullerton, CA). Data analysis was performed using ImmunoFit EIA/RIA software (Beckman Instruments Inc). Standard curves were fitted to a 4-parameter logistic function and melatonin levels were reported as either absolute or relative values.

### cDNA microarray production

Microarrays were constructed from two cDNA libraries that were generated from chick mRNA isolated during midday (ZT6) and midnight (ZT18) as described previously [25]. Approximately 4000 cDNA clones from each library (8113 total) are represented in our custom microarray.

### Microarray hybridizations

Total RNA was extracted from cell lysates using a Qiagen RNeasy kit (Qiagen, Valencia, CA), then amplified using a MessageAmp II RNA amplification kit (Ambion, Austin, TX). Both total RNA and aRNA samples were analyzed on an Agilent 2100 Bioanalyzer for quantitation and quality control. cDNA was synthesized from randomly primed aRNA using a 3 DNA Array 350 RP kit (Genisphere, Hatfield, PA) and Superscript II RT-PCR enzyme and reagents (Invitrogen). cDNAs were then modified, concentrated, and hybridized to the array as recommended in the Genisphere users' protocol. Bound cDNA from each timepoint in both the LD and DD cultures were hybridized to Cy5 probes, while cDNA from samples collected at ZT18 or CT18 (from LD and DD cultures, respectively) were hybridized to Cy3 probes, and served as the control for each time series. For the light pulse experiment, cDNA from cells exposed to a light pulse was hybridized to Cy5 probes, while cDNA from control cells was hybridized to both Cy3 and Cy5 probes. Labeling was carried out in the same way for the norepinephrine experiment, where cDNA samples from norepinephrine treated cells served as the experimental channel, and samples that did not receive norepinephrine served as the control channel. As an additional control, dye swaps were carried out for cDNA samples in both the light pulse and norepinephrine experiments.

All hybridizations were carried out in SDS-based buffer, and slides were washed and dried following each hybridization as recommended (Genisphere). Slides were scanned for Cy5 and Cy3 fluorescence using an Affymetrix 428 array scanner, and .tif images were generated from scans for both channels. All microarray hybridizations were performed twice (N = 2 sample replicates) for each experimental group (N = 4 biological replicates), giving a total number of 8 replicates for all samples.

### Microarray analysis

The .tif images generated from the scanner were analyzed using GenePixPro (Axon Instruments, Union City, CA) to determine signal and background fluorescence, and a false color image was then generated for each dye. This application was then used to generate .gpr files, which were analyzed using GeneSpring (Silicon Genetics, Palo Alto, CA). Data from the LD and DD series (N = 8 per timepoint) were subjected to LOWESS normalization, and each time-point was reported as the normalized ratio of Cy5 to Cy3 intensity, where the ZT/CT 18 time point (for LD and DD, respectively) was designated as the control for each time series. Thus, expression of each gene at a given time-point was reported in terms of relative abundance to its own expression at midnight.

We established a multilevel analysis with different stringencies to determine which genes showed rhythmic expression patterns at different amplitudes. All analyses were based on two criteria: fold-change, and statistically significant variation, of expression levels relative to ZT/CT18. Our first statistical method defined rhythmic expression as: 1) having a minimum 1.5-fold difference in expression levels for at least one time point relative to midnight; and 2) having a significantly different level of expression for one or more time-points relative to midnight, based on two-sample Students' t-test comparisons. Our second statistical method required that gene expression show an overall statistically significant variation over time based on ANOVA, as well as exhibiting at least a 1.5-fold change in expression levels. In addition, we screened genes that met a 2-fold change requirement using both statistical methods. All filters based on fold-change were performed using a linear ratio interpretation, whereas all statistical filters were based on a log ratio interpretation within the GeneSpring program.

Analysis of the light pulse and norepinephrine dosage experiments (N = 8 per time-point per treatment) was done similarly; however, we performed additional dye-swap normalization along with LOWESS normalization for these data sets. In these experiments, Cy5 to Cy3 normalized experimental treatments (samples that had received light or NE) were compared to control samples using filters on statistical differences and fold change. Light or NE was considered to have an effect on gene expression if: 1) there was a minimum 1.5 fold difference between experimental and control treatments at CT6 or CT18; and 2) the difference was statistically significant based on a t-test. We also examined genes which showed 2-fold or greater regulation by light or norepinephrine. The data discussed in this publication have been deposited in NCBIs Gene Expression Omnibus (GEO, ) and are accessible through GEO Series accession number GSE5292.

### Quantitative real-time PCR analysis

Expression of selected genes from the microarray analysis was validated using quantitative real-time PCR (qPCR), as follows. Pineal culture aRNA was DNase treated, primed with random hexamers, and cDNA was synthesized by reverse transcription using a Superscript II RT PCR kit (Invitrogen). Relative quantitation of selected genes was achieved by performing SYBR green-based real-time PCR using an ABI Prism 7700 Sequence Detection instrument (Applied Biosystems, Foster City, CA). Primers optimized for SYBR green real-time PCR amplification were designed for selected genes using Primer Express (Applied Biosystems). Primer sequences are listed in Additional file 8.

Standard curves were generated for target gene cDNAs and for *cyclophilin*, which we used as an endogenous reference, and cDNA for each timepoint was run in triplicate for each plate. Target gene expression levels were normalized to the endogenous reference values, and then normalized to a calibrator sample, which consisted of a mix of cDNA from each timepoint. Each plate included a "no template control" reaction (cDNA was replaced with water) as well as an "RT-control" reaction (reverse transcriptase enzyme was replaced with water) to rule out the possibility of genomic contamination.

### Statistical analysis

Time course data for microarray validation were subjected to cosinor analysis utilizing linear harmonic regression (CircWave software) [40], as well as ANOVA. Changes in melatonin levels (light and NE experiments) were subjected to a two-sample t-test. ANOVA and t-tests were performed using Sigma Stat software package (Systat Software Inc, Point Richmond, CA).

## Authors' contributions

SPK drafted the manuscript and carried out the cell cultures, sampling, radioimmunoassays, microarray hybridizations, microarray validations by real time PCR, statistics, and all data analyses. VK helped develop and adapt the radioimmunoassay methods, and assisted with cell culture, sampling, and performing microarray hybridizations. PDB oversaw the development and printing of custom microarray slides and helped with the microarray analysis. MJB helped develop the cDNA library and construct the custom microarrays. TLT and VMC oversaw the design of the study, provided critical intellectual contributions, and helped revise the manuscript.

All authors read and approved the final manuscript.

## Supplementary Material

Additional file 1 **Rhythmic transcripts (t-test analysis)**. This file lists gene transcripts determined to be rhythmic using t-test analysis. Separate tabs contain lists of gene transcripts which exhibit rhythm amplitudes of at least 1.5-fold or 2-fold in either LD or DD. Lists are presented as two columns of data displaying each clone ID and associated reference number as listed in the Texas A&M Biology Department's Laboratory for Functional Genomics chicken pineal database [32]. Data are listed in alphabetical order by clone ID. Click here for file

Additional file 2 **Rhythmic transcripts (ANOVA)**. This file lists gene transcripts determined to be rhythmic using ANOVA. Separate tabs contain lists of gene transcripts which exhibit rhythm amplitudes of at least 1.5-fold or 2-fold in either LD or DD. Lists are presented as two columns of data displaying each clone ID and associated reference number. Data are listed in alphabetical order by clone ID. Click here for file

Additional file 3 **Functional clustering of rhythmic transcripts**. This file lists functional categorizations of non-redundant gene transcripts determined to be rhythmic using t-test analysis. Separate tabs contain lists of gene transcripts which rhythmic in either LD or DD. Lists are presented as two columns of data displaying each clone ID and functional cluster. Data are listed in alphabetical order by clone ID. Click here for file

Additional file 4 **Light regulated transcripts**. This file lists gene transcripts regulated by 6-hour exposure to light. Separate tabs contain lists of gene transcripts that were upregulated or downregulated at least 1.5-fold or 2-fold at either CT6 or CT18. Lists are presented as two columns of data displaying each clone ID and associated reference number. Data are listed in alphabetical order by clone ID. Click here for file

Additional file 5 **NE regulated transcripts**. This file lists gene transcripts regulated by 6-hour exposure to norepinephrine. Separate tabs contain lists of gene transcripts that were upregulated or downregulated at least 1.5-fold or 2-fold at either CT6 or CT18. Lists are presented as two columns of data displaying each clone ID and associated reference number. Data are listed in alphabetical order by clone ID. Click here for file

Additional file 6 **Comparative analysis**. This file contains non-overlapping unigene lists, clustered as follows: LD: rhythmic genes with at least 1.5-fold amplitude mRNA expression in LD; DD: rhythmic genes with at least 1.5-fold amplitude mRNA expression in DD; Light: gene mRNA regulated at least 1.5-fold by light; NE: gene mRNA regulated at least 1.5-fold by norepinephrine. Data are listed in alphabetical order by clone ID. Genes for which returned no blast hit was returned are listed by reference number. Click here for file

Additional file 7 **Candidate genes**. This file contains a brief discussion of several candidate genes from the genomics screen. Click here for file

Additional file 8 **Primer sequences**. This table lists primer sequences for all qPCR targets. Click here for file
