# Supplementary material for: Circadian genomics of the chick pineal gland in vitro
Source: BMC Genomics. 2008 May 3;9:206. doi: 10.1186/1471-2164-9-206 (PMC2405806; doi:10.1186/1471-2164-9-206)
Supplement: Additional file 7 — Candidate genes. This file contains a brief discussion of several candidate genes from the genomics screen. [file 1471-2164-9-206-S7.doc]

| qPCR primer | Primer Sequence (5’-3’) |
| --- | --- |
| Cry1-F | CCGGGAAACGCCCAAA |
| Cry1-R | TGCTCTGCCGCTGGACTT |
| Cyclophilin-F | GCAAGCAGATCACCATTTCCA |
| Cyclophilin-R | CGGAATGTCAGGCGTTAAGAC |
| Cystatin C-F | GAACGACGAGGGCTTGCA |
| Cystatin C-R | TTATCGTTGCTGGCCCTGTT |
| HIOMT-F | TGCAGTCAGGACGACCTCTATCT |
| HIOMT-R | TCCATCCCCATGATGCTTGT |
| NF1X-F | TGATACCGCCGCCATGTAC |
| NF1X-R | GCCTCGATGAAGGGATGGA |
| Per3-F | CAGAATGGAAACGATCAGCCTAT |
| Per3-R | TCGGGAGAAAACAGGAAGCA |
| Purpurin-F | TGCTGACATGGCTGCTCAGT |
| Purpurin-R | CCCTGGTACGTCATGTACATCTT |
| TrH-F | GCCGAGGGCTTGTAAAAACTC |
| TrH-R | GAATTCCGGGACCGCAAT |
