# Supplementary material for: Circadian genomics of the chick pineal gland in vitro
Source: BMC Genomics. 2008 May 3;9:206. doi: 10.1186/1471-2164-9-206 (PMC2405806; doi:10.1186/1471-2164-9-206)
Supplement: Additional file 8 — Primer sequences. This table lists primer sequences for all qPCR targets. [file 1471-2164-9-206-S8.doc]

***NF1X***

*Nuclear factor 1 X-type* (*NF1X*) is a transcription factor known to bind the palindromic consensus sequence TTGGC(N)_5_GCCAA [1], and has been shown to activate replication of adenoviral DNA [2]. It is highly conserved in vertebrates, with chicken and hamster orthologs showing 92% amino acid sequence identity [3]. *NF1X* is reported to control the expression of a number of different genes in liver [4-8], and is a known repressor of *glutathione S-transferase* [9], which is involved in intermediary metabolism of xenobiotics and is also shown to be rhythmic in our study (additional file 1). NF1 proteins also exhibit a redox-sensitive regulation of *CYP1A* transcription in humans [10]. Since CYP1A protein levels alter the oxidative state of the cell, which in turn activates the transcription of multiple transcription factors [11], *CYP1A* could provide a direct link between the pineal clock, cellular redox state, and intermediary metabolism if the circadian clock regulated it. Future research exploring a redox dependent regulatory role of *NF1X* within the chick pineal clock is warranted.

# *Cystatin C*

C*ystatin* is a potent cysteine protease inhibitor [12] and has been implicated in diverse processes, including immunomodulation. Chicken *cystatin* has also been reported to act as a growth hormone in mouse fibroblasts [12]. The putative role of *cystatin* in immune function is intriguing, given that many genes associated with the immune system show circadian rhythmicity *in vivo* [13] as well as *in vitro* (Fig. 2). Furthermore, we hypothesize that cystatin may interact with redox-sensitive pathways at the posttranslational level, since cysteine thiol groups are the primary redox-sensing structures.

## *NDRG1*

*N-myc downstream regulated 1* (*NDRG1*) is involved in a wide array of biological processes, including cellular differentiation and stress responses [14, 15], and is repressed by the *n-myc* and *c-myc* proto-oncogenes. The rhythmic and light inducible expression of *NDRG1* may indicate circadian regulation of *n-myc* itself. This finding would be of interest since n-myc protein activates transcription via binding to E-boxes [16], and subsequently the activation of a large number of genes involved in ribosomal and protein synthesis [17], consistent with the result of our functional clustering analysis of pinealocytes *in vitro*.

Additionally, *NDRG1* is reported to be induced by retinoic acid [15] and to associate with apolipoprotein A-I [18]. apolipoprotein A-I is a gene product involved in cholesterol transport which we found to be regulated in a circadian fashion within the chick pineal (additional file 6). *NDRG1* may therefore couple retinoic acid signaling with circadian regulation of cellular trafficking of lipids in the pineal.

## *Purpurin*

*Purpurin* belongs to the lipocalin protein family, a diverse group of proteins involved in various processes including immune function and retinoid binding [19]. *Purpurin* is known to be synthesized in retinal photoreceptors [20], and while its function is not fully understood, it is thought to mediate cellular adhesion and survival [21] as well as having a role in the transport of retinol within the retina [22]. These properties make *purpurin* an interesting potential candidate gene for linking visual input or immune response to the pinealocyte clock.

**REFERENCES**

1. Nowock J, Borgmeyer U, Puschel AW, Rupp RA, Sippel AE: **The TGGCA protein binds to the MMTV-LTR, the adenovirus origin of replication, and the BK virus enhancer.** *Nucleic Acids Res* 1985, **13**:2045-2061.

2. Nagata K, Guggenheimer RA, Hurwitz J: **Specific binding of a cellular DNA replication protein to the origin of replication of adenovirus DNA.** *Proc Natl Acad Sci* *USA* 1983, **80**:6177-6181.

3. Kruse U, Oian F, Sippel AE: **Identification of a fourth nuclear factor 1 gene in chicken by cDNA cloning: NF1X** *Nucleic Acids Res* 1991, **19**:6641.

4. Anania F, Potter J, Rennie-Tankersley L, Mezey E: **Effects of acetaldehyde on nuclear protein binding to the nuclear factor I consensus sequence in the alpha 2(I) collagen promoter.** *Hepatology* 1995, **21**:1640-1648.

5. Lichtsteiner S, Wuarin J, Schibler U: **The interplay of DNA-binding proteins on the promoter of the mouse albumin gene.** *Cell* 1987, **51**:963-973.

6. Garlatti M, Aggerbeck M, Bouguet J, Barouki R: **Contribution of a nuclear factor 1 binding site to the glucocorticoid regulation of the cytosolic aspartate aminotransferase gene promoter.** *J Biol Chem* 1996, **271**:32629-32634.

7. Bois-Joyeux B, Danan JL: **Members of the CAAT/enhancer-binding protein, hepatocyte nuclear factor-1 and nuclear factor-1 families can differentially modulate the activities of the rat alpha-fetoprotein promoter and enhancer.** *Biochem J* 1994, **301**:49-55.

8. Cardinaux JR, Chapel S, Wahli W. **Complex organization of CTF/NF-I, C/EBP, and HNF3 binding sites within the promoter of the liver-specific vitellogenin gene.** *J Biol Chem* 1994, **269**:32947-32956.

9. Osada S, Ikeda T, Xu M, Nishihara T, Imagawa M: **Identification of the transcriptional repression domain of nuclear factor 1-A.** *Biochem Biophys Res Commun* 1997, **238**: 744-747.

10. Morel Y, Barouki R: **Down-regulation of cytochrome P450 1A1 gene promoter by oxidative stress—critical contribution of nuclear factor 1.** *J Biol Chem* 1998, **273**: 26969-26976.

11. Puga A, Barnes SJ, Chang C, Zhu H, Nephew KP, Khan SA, Shertzer HG: **Activation of Transcription Factors Activator Protein-1 and Nuclear Factor-kB by 2,3,7,8-Tetrachlorodibenzo-*p*-dioxin.** *Biochem Pharm* 2000, **59**:997-1005.

12. Dickinson DP: **Salivary (SD-type) cystatins: over one billion years in the making—but to what purpose?** *Crit Rev Oral Biol Med* 2002, **13**:485-508.

13. Bailey MJ, Beremand PD, Hammer R, Bell-Pedersen D, Thomas TL, Cassone VM: **Transcriptional profiling of the chick pineal gland, a photoreceptive circadian oscillator and pacemaker.** *Mol Endocrinol* 2003, **17**:2084-2095.

14. Agarwala KL, Kokame K, Kata H, Miyata T: **Phosphorylation of RTP, an ER stress-responsive cytoplasmic protein.** *Biochem Biophys Res Commun* 2000, **272**:641-647.

15. Piquemal D, Joulia P, Balaguer A, Basset A, Marti J, Commes T: **Differential expression of the RTP/Drg1/Ndr1 gene product in proliferating and growth arrested cells.** *Biochim Biophys Acta* 1999, **1450**:364-373.

16. Alex R, Sozeri O, Meyer S, Dildrop R: **Determination of the DNA sequence recognized by the bHLH-zip domain of the N-myc protein.** *Nucleic Acids Res* 1992, **20**:2257-2263.

17. Boon K, Caron HN, van Asperen R, Valentijn L, Hermus MC, van Sluis P, Roobeek I, Weis I, Voute PA, Schwab M, Versteeq R: **N-myc enhances the expression of a large set of genes functioning in ribosome biogenesis and protein synthesis.** *The EMBO Journal* 2001, **20**:1383-1393.

18. Hunter M, Angelicheva D, Tournev I, Ingley E, Chan DC, Watts GF, Kremensky I, Kalaydjieva L: **NDRG1 interacts with APO A-I and A-II and is a functional candidate for the HDL-C QTL on 8q24.** *Biochem Biophys Res Commun* 2005, **332**:982-92.

19. Flower DR: **The lipocalin protein family: a role in cell regulation.** *FEBS Lett* 1994, **354**:7-11.

20. Berman P, Gray P, Chen E, Keyser K, Ehrlich D, Karten H, LaCorbiere M, Esch F, Schubert D: **Sequence analysis, cellular localization, and expression of a neuroretina adhesion and cell survival molecule.** *Cell* 1987, **51**:135-142.

## 21. Schubert D, LaCorbiere M: **Isolation of an adhesion-mediating protein from chick neural retina adherons.** *J Cell Biol* 1985, **101**:1071-1077.

22. Schubert D, LaCorbiere M, Esch F: **A chick neural retina adhesion and survival molecule is a retinal-binding protein.** *J Cell Biol* 1986, **102**:2295-2301.
